# Survival, Recorded Disability, and Neurorehabilitation After Advanced Endovascular Stroke Care in Kazakhstan: A Retrospective Cohort Study

**DOI:** 10.3390/jcm15155964

**Published:** 2026-07-30

**Authors:** Akerke Alibekovna Toleu, Mohammad Khoshraftar, Aizhan Bolatovna Almukhanova, Lyudmila Sergeevna Yermukhanova, Ardak Nurbakytovna Nurbakyt, Yesbol Tynysbekovich Toleu, Maral G. Nogayeva, Alireza Afshar

**Affiliations:** 1 Department of Public Health and Healthcare, West Kazakhstan Marat Ospanov Medical University, Aktobe 030000, Kazakhstan; a.toleu@zkmu.kz; 2 Student Research Committee, Bushehr University of Medical Sciences, Bushehr 7514633341, Iran; khoshraftarm.mk@gmail.com; 3 Department of Internal Medicine, Asfendiyarov Kazakh National Medical University, Almaty 050012, Kazakhstan; aizhanbolat84@gmail.com; 4 Department of Public Health, Asfendiyarov Kazakh National Medical University, Almaty 050012, Kazakhstan; a.nurbakyt@kaznmu.kz; 5 Almaty City Branch, S. Kairbekova National Scientific Center for Health Development, Konaev 040800, Kazakhstan; e.toleu@nrchd.kz; 6 Department of Rheumatology, Asfendiyarov Kazakh National Medical University, Almaty 050012, Kazakhstan

**Keywords:** stroke, cerebrovascular stroke, thrombectomy, stents, rehabilitation, survival analysis

## Abstract

**Background**: Advanced endovascular procedures are increasingly used for ischemic stroke, but real-world evidence from middle-income settings remains limited, particularly regarding survival, disability, and neurorehabilitation. This study examined mortality, recorded disability, procedure-related differences, and neurorehabilitation exposure among patients receiving high-technology stroke care in Almaty, Kazakhstan. **Methods**: We conducted a retrospective cohort study of 328 adults with ischemic stroke who underwent endovascular or stent-based procedures between 2014 and 2024. Survival was assessed using Kaplan–Meier analysis and Cox proportional-hazards regression. Binary mortality status and recorded disability were examined using logistic regression. Procedure codes were grouped as mechanical thrombectomy/endovascular removal, intracranial stenting, and embolization/coiling/other procedures. **Results**: Neurorehabilitation was received by 39% of patients and was associated with better crude survival (1 year survival 93.6% vs. 76.7%; 5-year survival 69.0% vs. 60.7%; log-rank *p* = 0.001). In the adjusted Cox model, older age (HR = 1.069, 95% CI 1.046–1.092, *p* < 0.001) and greater total bed-day burden (HR = 1.071, 95% CI 1.029–1.116, *p* = 0.001) were associated with higher mortality hazard. Binary neurorehabilitation was not independently associated with survival (*p* = 0.803). Male sex was the only consistent factor associated with recorded disability (fully adjusted OR = 2.662, 95% CI 1.261–5.617, *p* = 0.010). **Conclusions**: Older age and bed-day burden were the most stable mortality-related factors. Neurorehabilitation was linked to better crude survival but not adjusted survival or recorded disability. Procedure-related and disability findings should be considered exploratory because baseline stroke severity and validated functional scales were unavailable.

## 1. Introduction

Stroke is the second leading cause of mortality and third leading cause of disability worldwide, causing about seven million deaths annually and producing around 12 million new strokes each year [1,2]. Approximately two-thirds of strokes are ischemic, and more than 87% of deaths and 89% of disability adjusted life years (DALYs) occur in low- and middle-income countries (LMICs) [1]. These stark statistics highlight the imperative for effective prevention, timely acute management and comprehensive rehabilitation [3].

The substantial burden of stroke is largely preventable [2]. The Global Burden of Disease study attributes around 84% of stroke DALYs to modifiable risk factors, chiefly hypertension, diabetes, obesity and unhealthy lifestyles [1,2]. Metabolic, behavioral and environmental risks collectively contribute to the burden [1,4]. Population-level control of blood pressure and promotion of healthy living are therefore essential, yet many patients still experience acute stroke. Improving acute treatment and post-stroke care is crucial, particularly in LMICs where specialized services remain limited [1,3].

Regional differences in the distribution of risk factors and access to care highlight the necessity of context-specific prevention strategies [5]. In many LMICs, health systems face dual burdens of infectious and chronic diseases and limited infrastructure for early detection and management of hypertension and diabetes [6,7]. As a result, prevention must be coupled with robust acute care and rehabilitation services to reduce stroke mortality and disability [8].

The advent of high-tech endovascular therapies has transformed acute ischemic stroke care. Mechanical thrombectomy, which removes an occluding thrombus through an endovascular approach, has become a standard treatment for eligible patients with large-vessel occlusion [9,10]. In a recent emulation of randomized trials, patients receiving thrombectomy achieved higher rates of functional independence and lower mortality than those receiving thrombolysis alone. These findings, corroborated by earlier trials, demonstrate that mechanical thrombectomy combined with best medical therapy improves outcomes compared with thrombolysis alone [9,10,11]. Guidelines recommend thrombectomy within 6 to 24 h of stroke onset using imaging selection, but timely access depends on specialized teams and infrastructure that are often lacking in LMICs [2].

By contrast, the role of intracranial stenting for stroke prevention remains controversial [3]. Early trials such as SAMMPRIS and VISSIT reported high rates of stroke or death with stenting compared with medical therapy [3,12]. Subsequent series using tailored approaches have reported lower complication rates and improved outcomes. These mixed results suggest that outcomes may improve with refined techniques, but robust evidence is limited and high-tech interventions are resource intensive [13,14]. Because outcomes depend heavily on vascular anatomy and operator experience, careful patient selection and procedural expertise are critical to maximizing benefits and minimizing risks when considering stenting [3,12,14].

Long-term functional recovery is as important as survival [15,16]. Neurorehabilitation aims to enhance recovery and reduce disability [15]. A meta-analysis of randomized trials reported that very early mobilization (within 48 h) improved activities of daily living and limb motor function and reduced long-term complications, but overly aggressive mobilization may increase mortality [15,16]. Experimental studies suggest that early exercise promotes repair by modulating inflammatory cytokines. These findings underscore the potential synergy between advanced interventions and timely rehabilitation [15,16]. Further research is required to determine the most effective timing, intensity and composition of rehabilitation programmes after endovascular procedures and to understand how these interventions interact with advanced therapies in different clinical contexts [15,16].

Despite technological advances, major gaps remain. Evidence on high-tech stroke interventions largely derives from high-income countries, while LMICs, where the majority of stroke burden occurs, have limited data on survival, disability and resource utilization [1,4]. Moreover, the epidemiological context and health-care infrastructure in middle-income countries differ markedly from those in high-income settings, so extrapolating results from the latter may not fully reflect outcomes in resource-constrained environments. By examining endovascular stroke care in Almaty, Kazakhstan, our study provides region-specific data that contribute to the scarce real-world evidence from middle-income countries and can inform local policy and practice [17,18]. Access to endovascular services and neurorehabilitation is often restricted in LMICs, and outcomes may differ due to delays in presentation, comorbidities and infrastructure constraints. Moreover, most studies evaluate mechanical thrombectomy or stenting in isolation and rarely assess long-term functional outcomes or the interplay between neurorehabilitation and high-tech interventions [3,4,10]. Systematic evaluations of survival and disability after advanced procedures in resource-limited settings are scarce. Filling these gaps is essential for policymakers to prioritize resources and develop evidence-based stroke care pathways [19,20].

Shortages of interventional neuroradiologists and rehabilitation specialists in LMICs further hinder implementation of high-tech procedures and comprehensive post-stroke care [21]. Enhanced data on how resource constraints influence survival and disability will inform strategic planning and resource allocation. Hence, rigorous evaluation of outcomes in such settings is vital to ensure equitable stroke care [22,23].

Thus, this study aims to describe survival and disability outcomes after high-tech medical care for ischemic stroke and examine associations with neurorehabilitation in a resource-limited setting. We conducted a retrospective cohort study of 328 adults treated with advanced endovascular procedures, percutaneous intracranial stent insertion, endovascular embolization or occlusion, endovascular removal of obstruction and coil embolization, at stroke centers in Almaty, Kazakhstan. We examined demographics, admission routes, procedure types, hospital-stay metrics and participation in neurorehabilitation. Our primary objective was to assess long-term survival and recorded disability status using Kaplan–Meier survival curves and Cox proportional-hazards modelling. Secondary objectives included investigating whether neurorehabilitation and hospital utilization were associated with outcomes. By analyzing real-world data from a middle-income country, we aim to inform clinicians and policy makers about the benefits and limitations of high-tech interventions, identify determinants of poor outcomes and highlight areas for future research.

## 2. Materials and Methods

### 2.1. Study Design and Setting

We conducted a retrospective cohort study at stroke centers in Almaty, Kazakhstan. The cohort comprised 328 adults with ischemic stroke who underwent an endovascular or stent-based procedure between January 2014 and December 2024.

### 2.2. Participants and Data Sources

We reviewed inpatient records of 328 adult patients (≥18 years) who underwent high-tech procedures for a confirmed diagnosis of ischemic stroke (ICD-10 code I63). Records were obtained from regional stroke centers authorized to perform endovascular interventions. Patients were included if they had complete documentation of demographics, treatment details, survival status and functional outcomes. Patients with hemorrhagic stroke (ICD-10 codes I61-I62), those younger than 18 years, or those who did not receive an interventional procedure were excluded. Ethics approval was obtained from the university’s institutional review board, and all data were anonymized prior to analysis.

### 2.3. Variables and Measurements

Demographic information (age, sex, place of residence) and clinical variables (admission route, ICD-9-CM procedure code, inpatient and total bed-days) were extracted from charts and entered into a standardized Excel database. Inpatient bed-days were counted from the procedure date to discharge from the acute-care hospital, whereas total bed-days encompassed the sum of days spent in intensive care, intermediate care, and rehabilitation wards during the index hospitalization. High-tech procedures were classified using ICD-9-CM procedure codes: percutaneous intracranial vascular stent insertion (00.65), endovascular embolization/occlusion (39.72), endovascular removal of obstruction (39.74), and embolization using bare coils (39.75). Codes 39.72 and 39.75, which commonly indicate embolization or coiling procedures for aneurysmal subarachnoid hemorrhage, were included because these interventions were coded under ischemic stroke in our hospital database. We recognize that these cases may represent heterogeneous pathologies; therefore, their outcomes are interpreted descriptively and considered separately where appropriate. We grouped individual procedure codes into three categories: mechanical thrombectomy/endovascular removal, intracranial stenting, and embolization/coiling/other procedures, to enhance statistical power and because these interventions share similar vascular access techniques and acute-care workflows [17,19]. Key clinical variables such as the National Institutes of Health Stroke Scale, ASPECTS scores, occlusion site, infarct burden, onset-to-treatment time, reperfusion success, hemorrhagic complications, and comorbidities were unavailable in the administrative dataset; therefore, we could not adjust for baseline severity or confounding by indication [19]. Recorded disability refers to the final documented disability status at discharge or during follow-up, as captured in clinical or administrative records. Because standardized functional scales such as the modified Rankin Scale or Barthel Index were not routinely captured in our hospital records, disability status was obtained from chart-based clinical assessments and legal disability certification, which may reflect both functional impairment and socio-economic factors in Kazakhstan. We adopted a binary classification to maximize consistency across cases and to align with the dichotomous disability certification used for social support in our setting [19]. Receipt of neurorehabilitation was coded as yes/no, and the number of neurorehabilitation hospitalizations was also recorded. Survival time was calculated as the number of days from the date of procedure to death or last follow-up; patients alive at last follow-up were censored. Therefore, the variable reported as ‘time in days until death’ represented the duration between the procedure and last follow-up; for patients alive at last follow-up, it corresponded to censoring time rather than days to death.

### 2.4. Outcomes

The primary outcome was overall survival (time to death). Secondary outcomes included binary survival status (deceased vs. alive at follow-up) and recorded disability, defined as the final documented disability status at discharge or during follow-up. Neurorehabilitation participation and hospital utilization measures were assessed as explanatory variables of these outcomes.

### 2.5. Sample Size and Sampling Method

The original proposal anticipated a sample of approximately 110 patients based on prevalence estimates and a 5% margin of error. For the final analysis, however, all 328 eligible patients were included to maximize statistical power and representativeness. This complete enumeration approach aligns with the proposal’s emphasis on studying the full volume of high-tech procedures performed in Almaty during the study period.

### 2.6. Statistical Analysis

Data analyses were performed using SPSS Version 26.0 (IBM Corp., Armonk, NY, USA). Continuous variables were summarized as means ± standard deviations and compared using independent-samples t tests or one-way ANOVA, as appropriate. Categorical variables were presented as frequencies and percentages and compared using χ^2^ tests or Fisher’s exact test. Kaplan–Meier curves were used to compare crude survival according to neurorehabilitation status and procedure group, and log-rank tests were used to assess differences between survival curves. Multivariable Cox proportional-hazards regression was used to identify factors associated with time-to-death, adjusting for age, sex, neurorehabilitation, number of neurorehabilitation hospitalizations, inpatient bed-days, total bed-days, and clinically grouped procedure type. Dates of rehabilitation initiation were unavailable; therefore, neurorehabilitation was treated as a fixed covariate, which introduces potential immortal time and survivor bias, and the resulting hazard ratios should not be interpreted causally [19]. Binary logistic regression was used as a supplementary analysis of final mortality status and as the main multivariable analysis for recorded disability. For the disability model, time-to-death was not included as a predictor to avoid survival-dependent interpretation. Procedure-specific exploratory analyses were performed within clinically grouped procedure strata, but these were interpreted cautiously because some subgroups contained few mortality or disability events and several models did not converge. Statistical significance was defined as a two-sided *p* < 0.05. Because event counts were limited in some models, estimates were interpreted with attention to confidence intervals, model convergence, and events-per-variable constraints [24,25].

## 3. Results

### 3.1. Older Age, EMS Admission, and Longer Hospital Stay Were Associated with Higher Mortality, While Male Sex Was Associated with Greater Disability

The cohort included 328 patients who underwent advanced endovascular interventions for ischemic stroke. The mean age of the cohort was 63.4 ± 11.8 years, but those who died were significantly older (69.8 ± 9.3 years) than survivors (61.2 ± 11.8 years; *p* < 0.001, Table 1). Disability at the end of follow up showed a non-significant trend toward being younger (60.5 ± 10.5 vs. 63.9 ± 12.0 years; *p* = 0.071). Sex distribution did not differ between deceased and living patients (61.6% male vs. 38.4% female; *p* = 0.451), but disability was more common in men (18.3% vs. 8.7%, *p* = 0.017). Urban and rural residence showed no differences in survival or disability.

Admission type was associated with survival (χ^2^, *p* = 0.035): patients arriving via emergency medical services (EMS) had higher mortality (28.5%) than self referrals (11.7%) or primary health care admissions (21.4%). Admission type did not predict disability. Among procedure codes, percutaneous intracranial stenting (00.65) and endovascular removal of obstruction (39.74) were most common (31.1% and 61.0%, respectively). Mortality was lower after stenting (14.7% vs. 30.5% for 39.74), but the distribution across codes differed significantly only for survival (*p* = 0.021). Mean inpatient stay was longer in deceased patients (15.6 ± 10.3 days) than survivors (12.2 ± 6.4 days; *p* < 0.001), and total bed days showed the same pattern. Time to death averaged 525 days in deceased patients versus 967 days in survivors (*p* < 0.001) and was significantly longer in those who became disabled (1263 ± 962 days) than in those who remained functional (785 ± 682 days; *p* < 0.001).

### 3.2. Neurorehabilitation Was Associated with Lower Crude Mortality but Was Not Associated with Recorded Disability

Neurorehabilitation was delivered to 39% of patients; the remaining 61% did not receive it (Table 2). Age, sex and admission type were similar between groups, with mean ages of 62.6 ± 10.7 and 63.9 ± 12.5 years (*p* = 0.323). Urban residents were more likely to receive neurorehabilitation (40.8% vs. 20.7% of rural patients, *p* = 0.034). Procedure codes were evenly distributed: 35.3% of the neurorehabilitation group received code 00.65 and 42.0% received code 39.74, similar to the non-rehabilitation group. Inpatient and total bed days, as well as time to death, did not differ between groups.

Clinical outcomes differed markedly: mortality was lower among neurorehabilitation recipients (21.7%) than non-recipients (44.9%; *p* < 0.001). Disability rates were similar (37.5% vs. 39.3%, *p* = 0.815). Patients undergoing neurorehabilitation had more frequent rehabilitation hospitalizations (0.31 ± 0.7 vs. 0.12 ± 0.3; *p* = 0.005), indicating intensity of therapy rather than mere participation may be important.

### 3.3. Kaplan–Meier Analysis Showed Higher Crude Survival Probabilities in Patients Who Received Neurorehabilitation

**Figure 1:**
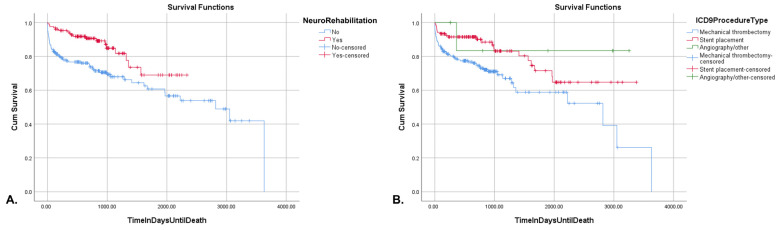
Kaplan–Meier survival curves by neurorehabilitation status (**A**) and neuro-intervention procedure type (**B**). (**A**) compares survival between patients who received neurorehabilitation and those who did not. (**B**) compares survival across ICD-9 procedure-type groups: Group 1 = mechanical thrombectomy/endovascular removal of obstruction, Group 2 = stent-based procedures, and Group 3 = other endovascular procedures. Time was measured from the index procedure to death or last follow-up; censored cases represent patients alive at last follow-up.

In the Kaplan–Meier analysis comparing patients who underwent neurorehabilitation with those who did not, the two survival curves diverged early and remained separated throughout follow up (Figure 1A). Among 128 patients who received neurorehabilitation and 200 who did not, the estimated survival at one year was 93.6% versus 76.7%, respectively, and at five years was 69.0% versus 60.7%. The median survival time was not reached in the neurorehabilitation group because the survival probability never fell to 50%, whereas in the non-rehabilitation group the median survival time was about 2 800 days. A log rank test, which compares the entire survival curves rather than survival at a single time point, showed a statistically significant difference between the groups (χ^2^ = 10.55, *p* = 0.001), associated with better long-term survival.

Kaplan–Meier survival analysis was also performed according to ICD-9 procedure type to explore whether survival differed across specific high-technology neuro-interventions (Figure 1B). Survival distributions differed significantly between procedure groups by the log-rank test, χ^2^ = 10.856, df = 2, *p* = 0.004. Patients in the stent placement group showed higher cumulative survival probabilities than those in the endovascular removal of obstruction/thrombectomy group, whereas the angiography/other group contained very few patients and should be interpreted cautiously. This analysis was exploratory and unadjusted; therefore, differences between procedure groups may reflect patient selection, baseline stroke severity, procedural indication, or other unmeasured clinical factors rather than the independent effect of the procedure itself.

### 3.4. Age, Intensive Bed-Day Use and Procedure Type Were Associated with Time to Death; Neurorehabilitation Was Not Independently Associated with Survival

A refined multivariable Cox model was constructed using clinically grouped procedure categories comprising mechanical thrombectomy/endovascular removal, intracranial stenting, and embolization/coiling/other procedures, while excluding sparse admission pathways. Overall model fit improved significantly compared with the null (Δχ^2^ = 104.77, df = 8, *p* < 0.001). Each additional year of age increased the hazard of death by approximately 7% (HR = 1.069, 95% CI 1.046–1.092, *p* < 0.001). Greater bed-day burden remained an independent risk factor; every additional unit of total bed-days, defined as the cumulative days spent in intensive care, intermediate care, and rehabilitation wards during the index hospitalization, was associated with a 7% higher mortality hazard (HR = 1.071, 95% CI 1.029–1.116, *p* = 0.001), whereas inpatient bed-days alone were not significant. Binary receipt of neurorehabilitation did not predict survival (HR = 1.148, 95% CI 0.388–3.403, *p* = 0.803), and the number of rehabilitation hospitalizations showed only a non-significant inverse association (HR = 0.554, 95% CI 0.287–1.068, *p* = 0.078), suggesting that the apparent survival benefit observed in crude analyses may be confounded by survivor bias and unmeasured stroke severity. Male sex exhibited a non-significant trend toward higher mortality (HR = 1.549, 95% CI 0.955–2.511, *p* = 0.076). Procedure group was not statistically significant overall (*p* = 0.094), but intracranial stenting displayed a lower mortality hazard compared with mechanical thrombectomy/endovascular removal (HR = 0.515, 95% CI 0.276–0.960, *p* = 0.037), while embolization/coiling/other procedures did not differ significantly from the reference group (HR = 0.650, 95% CI 0.276–1.529, *p* = 0.323). These findings highlight age and intensive bed-day use as robust predictors of mortality, while neurorehabilitation effects remain exploratory due to the absence of time-stamped rehabilitation data and stroke-severity measures (Table 3).

### 3.5. Binary Mortality Analysis Supported Age and Bed-Day Burden as Factors Associated with Death

A supplementary hierarchical logistic regression analysis was performed to examine final binary mortality status (Table S1). The findings were broadly consistent with the Cox model: older age and greater total bed-day burden were associated with higher odds of death in the fully adjusted model, while binary neurorehabilitation was not independently associated with mortality after full adjustment. Procedure group was not significant overall, although intracranial stenting showed lower odds of death compared with mechanical thrombectomy/endovascular removal in pairwise comparison. Because this model did not account for follow-up time, it was treated as a secondary analysis supporting the primary Cox regression.

### 3.6. Male Sex Was the Main Factor Associated with Disability After Endovascular Intervention

A hierarchical logistic regression analysis was performed to identify factors associated with disability status after endovascular treatment for ischemic stroke. In Model 1, which included age and sex, the model was statistically significant (χ^2^ = 9.113, df = 2, *p* = 0.010). Male sex was associated with higher odds of disability compared with female sex (OR = 2.339, 95% CI 1.141–4.794, *p* = 0.020), while age showed only a non-significant inverse trend (OR = 0.977, 95% CI 0.952–1.002, *p* = 0.077).

After adding living status and neurorehabilitation in Model 2, the model remained statistically significant (χ^2^ = 9.712, df = 4, *p* = 0.046). Male sex remained associated with disability (OR = 2.305, 95% CI 1.123–4.731, *p* = 0.023). In contrast, living status was not independently associated with disability (OR = 1.345, 95% CI 0.630–2.869, *p* = 0.443), and neurorehabilitation showed no association with disability (OR = 1.000, 95% CI 0.519–1.925, *p* = 0.999).

In the fully adjusted model, which additionally included inpatient bed-days, total bed-days, and clinically grouped procedure type, the overall model was marginal and did not reach conventional statistical significance (χ^2^ = 14.730, df = 8, *p* = 0.065; Nagelkerke R^2^ = 0.078). Only 48 disability events were available for eight predictors in the fully adjusted model, yielding approximately six events per variable; therefore, this model may be vulnerable to overfitting and unstable estimates. Male sex remained the only statistically significant factor associated with disability (OR = 2.662, 95% CI 1.261–5.617, *p* = 0.010). Age showed a non-significant inverse trend (OR = 0.974, 95% CI 0.948–1.001, *p* = 0.069), and total bed-days also showed a non-significant inverse trend (OR = 0.939, 95% CI 0.872–1.012, *p* = 0.092). Living status, neurorehabilitation, inpatient bed-days, and procedure type were not independently associated with disability. Compared with mechanical thrombectomy/endovascular removal, neither intracranial stenting nor embolization/coiling/other procedures differed significantly in the odds of disability.

Overall, the disability analysis indicated that male sex was the most consistent factor associated with disability in this cohort, whereas neurorehabilitation, procedure type, and hospital-stay measures were not independently associated with the binary disability outcome. Because the fully adjusted model was marginal and the number of disability events was limited, these findings should be interpreted as exploratory rather than predictive (Table 4).

### 3.7. Procedure-Specific Exploratory Analyses Showed Limited Stability Outside the Thrombectomy Subgroup

Several subgroup models were based on small numbers of events, and some supplementary models did not converge; therefore, these exploratory findings should be interpreted cautiously and viewed as hypothesis-generating rather than definitive. We focus on overall patterns and avoid overemphasizing subgroup-specific observations. Procedure-specific exploratory models were examined to assess whether the main findings were consistent across endovascular procedure groups (Tables S2 and S3). In the mechanical thrombectomy/endovascular removal subgroup, which contained the largest number of patients and events, the Cox model was statistically significant, and older age and greater total bed-day burden remained associated with higher mortality hazard. In contrast, neurorehabilitation, number of neurorehabilitation hospitalizations, inpatient bed-days, and sex were not independently associated with mortality within this subgroup. The disability model in the same subgroup was statistically significant, but its ability to classify disabled patients was limited, and only total bed-days reached statistical significance.

The intracranial stenting subgroup showed no statistically significant Cox model after adjustment, and no individual mortality predictor reached statistical significance. The corresponding disability model did not converge, indicating that the estimates were unstable and unsuitable for interpretation. In the embolization/coiling/other subgroup, the Cox model also failed to converge, and the disability model was non-significant with poor fit. These findings indicate that procedure-specific adjusted modelling was feasible mainly in the mechanical thrombectomy/endovascular removal group, whereas the smaller procedure strata were limited by sparse mortality and disability events. Therefore, stratified analyses were treated as exploratory and were not used for primary inference.

## 4. Discussion

### 4.1. Overview of Key Findings

This retrospective cohort included 328 adults who underwent advanced endovascular procedures for ischemic stroke in Almaty, Kazakhstan. Importantly, the disability endpoint was a binary status derived from clinical and administrative documentation rather than a validated functional measure such as the modified Rankin Scale or Barthel Index. It should therefore be interpreted as recorded disability and not as a direct measure of functional independence or post-stroke recovery. Older age and greater bed-day burden were the factors most consistently associated with mortality. Although crude Kaplan–Meier survival probabilities were higher among patients who received neurorehabilitation, neurorehabilitation was not independently associated with time to death in the adjusted Cox model. The overall procedure term was not statistically significant. Male sex was associated with recorded disability, but this finding was based on only 48 disability events and arose from a fully adjusted model that did not reach conventional statistical significance. The disability and procedure-related findings should therefore be considered exploratory.

### 4.2. Survival Outcomes and Predictors

Age was the factor most consistently associated with mortality in this cohort, with each additional year associated with an approximately 7% increase in the hazard of death. This finding is consistent with global stroke-burden data showing that stroke incidence, mortality, and disability increase substantially with aging populations [4]. In contrast, male sex did not reach statistical significance in the adjusted Cox model, although it showed a non-significant trend toward higher mortality. Therefore, sex-related mortality differences in this cohort should be interpreted cautiously and may reflect unmeasured comorbidities, health-seeking behavior, stroke severity, or social factors rather than an independent biological effect.

Total hospital bed-days were independently associated with mortality in the Cox model. This relationship may be bidirectional: longer stays could reflect greater stroke severity and complications, while extended hospitalization may predispose patients to hospital-acquired infections and deconditioning [26]. A large cohort from Brazil found that implementation of a stroke unit reduced the likelihood of prolonged stays by 43% but had no significant impact on mortality [27]. These results align with our findings, highlighting that efficient care pathways can shorten hospitalization without compromising survival. A study from Ethiopia reported that complications, aspiration pneumonia and hospital-acquired infections markedly increased mortality risk, whereas the use of antiplatelet therapy halved the hazard of death [26]. Although our dataset lacked detailed complication data, longer bed-days likely capture some of these adverse events. Because our dataset did not include stroke severity, comorbidities, post-stroke complications, medication use, or infection status, residual confounding remains likely. Accordingly, the association between greater bed-day burden and mortality may reflect underlying stroke severity, medical instability, or prolonged treatment of complications rather than a causal effect of hospitalization duration [28,29].

Although the overall procedure term was not statistically significant (*p* = 0.094), the pairwise comparison showed a lower mortality hazard for intracranial stenting than for mechanical thrombectomy/endovascular removal (HR = 0.515, 95% CI 0.276–0.960; *p* = 0.037). Because the procedure groups represented different clinical indications and baseline severity was unavailable, this pairwise association should not be interpreted as evidence of comparative effectiveness. Large randomized trials have historically demonstrated no net benefit of intracranial stenting over aggressive medical therapy; the SAMMPRIS and VISSIT trials reported higher 30-day stroke or death rates (14.7% and 24.1%) and no improvement in long-term outcomes. More recently, the CASSISS study showed improved procedural safety but still no difference in the risk of recurrent stroke [3,30]. Our finding of a possible survival advantage with stenting may reflect careful patient selection and evolving operator experience. Admission via emergency medical services was associated with higher crude mortality, which could indicate that sicker patients are transported by ambulance, whereas self-referral may capture milder strokes.

The role of neurorehabilitation requires cautious interpretation. Patients receiving neurorehabilitation had better crude survival in Kaplan–Meier analysis, but binary neurorehabilitation was not independently associated with mortality in the adjusted Cox model. The crude Kaplan–Meier survival difference should not be interpreted as evidence of a beneficial effect of neurorehabilitation because immortal time and survivor bias cannot be excluded [19,31]. The number of neurorehabilitation hospitalizations showed only a non-significant inverse association with mortality. This pattern suggests that rehabilitation exposure may be entangled with survivorship, referral practices, severity, and access to care. Trials and systematic reviews support the importance of rehabilitation for post-stroke recovery, but timing and intensity are critical, and very early or overly intensive mobilization may not benefit all patients [15,16]. Because our dataset lacked dates of rehabilitation initiation, session content, intensity, and outpatient rehabilitation data, the present findings should not be interpreted as evidence that neurorehabilitation either improves or fails to improve survival.

Despite multivariable adjustment, residual confounding remains likely because patients selected for neurorehabilitation may differ systematically from those not referred, including in stroke severity, functional reserve, socioeconomic status, referral patterns, and access to healthcare. Future prospective multicenter registries that collect baseline stroke-severity scores, standardized functional scales, time-stamped rehabilitation exposure, and detailed rehabilitation protocols could more robustly evaluate the causal impact of neurorehabilitation and validate our findings in similar settings [19,32].

### 4.3. Sex Differences in Recorded Disability

Male sex was associated with higher odds of recorded disability in the fully adjusted analysis (OR = 2.662, 95% CI 1.261–5.617). However, this finding should not be interpreted as evidence that men experienced poorer functional recovery. The outcome was derived from binary clinical or administrative documentation rather than a validated functional scale. Moreover, the overall fully adjusted model did not reach conventional statistical significance, and only 48 disability events were available for eight predictors. The observed association should therefore be regarded as exploratory.

Our finding differs from much of the existing stroke literature. Several cohort and registry studies have reported poorer unadjusted functional outcomes among women after stroke or mechanical thrombectomy. However, these differences frequently diminish after adjustment for age, pre-stroke disability, stroke severity, comorbidities, and reperfusion success. For example, a multicenter thrombectomy cohort found that women had lower unadjusted functional independence and higher mortality, but sex was not independently associated with outcome after adjustment for age, National Institutes of Health Stroke Scale score, pre-stroke modified Rankin Scale score, and reperfusion success [33]. Other registry studies have produced differing results, with some continuing to identify female sex as a factor associated with poorer functional status after multivariable adjustment [34]. These findings indicate that apparent sex differences in post-stroke outcomes are strongly influenced by clinical characteristics and the covariates included in the analysis.

Several factors may explain the different pattern observed in our cohort. First, our binary recorded-disability outcome may reflect administrative certification practices and socioeconomic criteria in addition to functional impairment. It is therefore not directly comparable with modified Rankin Scale or Barthel Index outcomes reported in other studies. Second, the dataset did not include stroke severity, pre-stroke functional status, infarct characteristics, cognition, mood, comorbidities, or reperfusion success. Residual confounding by these factors cannot be excluded. Finally, the limited number of recorded disability events increased the risk of overfitting and imprecise estimates. Prospective studies using standardized functional measures and comprehensive clinical adjustment are needed to determine whether sex-specific differences in post-stroke disability are present in this population [35].

### 4.4. Contextualizing Procedural and Rehabilitation Outcomes

In our multivariable Cox model, procedure category was not statistically significant overall (*p* = 0.094), although intracranial stenting showed a lower mortality hazard compared with mechanical thrombectomy/endovascular removal in pairwise comparison (HR = 0.515, 95% CI 0.276–0.960). In the disability model, procedure type was not independently associated with recorded disability. Because stroke-severity measures, including NIHSS, ASPECTS, infarct volume, pre-stroke functional status, occlusion site, onset-to-treatment time, reperfusion status, hemorrhagic complications, and comorbidities, were unavailable, associations between procedure type and mortality may be confounded by indication [36,37]. Patients selected for intracranial stenting are clinically distinct from those undergoing thrombectomy for acute large-vessel occlusion; therefore, grouping these procedures into broad categories reduces sparsity but does not eliminate clinical heterogeneity or residual confounding [19,32,38]. Interpretation of these findings requires caution for several reasons. First, procedure assignment was not randomized; patients selected for intracranial stenting may have had underlying atherosclerotic stenoses or tandem lesions that differ pathophysiologically from embolic large-vessel occlusions treated with thrombectomy alone. Second, our “stenting” group combined elective intracranial stenting for stenosis or aneurysm with rescue stenting after thrombectomy failure, while the “embolization/coiling/other” group was very small (n = 26), limiting statistical power. Third, procedure-specific exploratory models demonstrated that adjusted analyses were stable only in the mechanical thrombectomy subgroup; models in the stenting and embolization/coiling strata frequently failed to converge due to sparse events, reinforcing the need for cautious interpretation.

Existing literature on endovascular strategies provides context. A recent meta-analysis of nine studies comparing mechanical thrombectomy plus angioplasty or stent placement with thrombectomy alone for intracranial atherosclerotic large-vessel occlusion found no difference in 90-day mortality (risk ratio [RR] = 0.97, 95% CI 0.70–1.34, *p* = 0.84), but reported higher rates of functional independence (mRS 0–2) at 90 days in the angioplasty/stent group (RR = 1.24, 95% CI 1.11–1.38, *p* < 0.001) and reduced early neurological deterioration [39]. Another retrospective study comparing stent-assisted mechanical clot disruption with conventional intra-arterial thrombolysis for acute cerebral infarction showed better good-outcome rates in the stent group at 3 months (64% vs. 40%, *p* < 0.05) and 6 months (67% vs. 42%, *p* < 0.05), attributing this to shorter recanalization times [40]. However, more contemporary analyses report that adding stenting to thrombectomy does not significantly affect mortality or functional independence after adjustment for comorbidities and tPA use [41]. These mixed results highlight that differences in patient selection, underlying pathology, and procedural techniques influence outcomes. Our finding of a lower hazard with stenting should therefore be interpreted as a signal of heterogeneity rather than a definitive benefit. Prospective trials and large registries with detailed information on occlusion etiology, collateral status, recanalization success and stroke severity are needed to determine which patients may benefit from adjunctive stenting [39].

Finally, the small “embolization/coiling/other” group in our cohort precludes meaningful conclusions. Endovascular coiling is typically used for aneurysmal subarachnoid hemorrhage rather than ischemic stroke, suggesting that these cases may represent atypical clinical scenarios. Given the rarity of this category and the absence of standard stroke-severity measures, we presented the stratified analyses in the Supplementary Material and did not use them for primary inference. Overall, our results suggest that procedure type was not independently associated with survival or disability differences in this real-world cohort once patient factors and hospital-stay burdens are considered.

### 4.5. Limitations of Study

Several limitations should be considered. First, the retrospective design and reliance on hospital records may have introduced selection bias, information bias, and residual confounding. Second, key clinical variables were unavailable, including NIHSS, ASPECTS, occlusion site, infarct volume, reperfusion status, thrombolysis use, comorbidities, complications, pre-stroke functional status, and medication data. These factors are known to influence mortality and functional recovery after ischemic stroke and may partly explain the observed associations [38]. This absence of baseline severity adjustment is the central methodological limitation of the study, particularly for comparisons across procedure groups and neurorehabilitation exposure, and these comparisons should therefore be interpreted as descriptive associations rather than adjusted estimates of treatment effectiveness [36,38]. Third, neurorehabilitation was captured only as a binary variable and as the number of rehabilitation hospitalizations; dates of rehabilitation initiation, therapy intensity, content, adherence, outpatient rehabilitation, and community-based rehabilitation were not available. Consequently, time-dependent or landmark analyses of rehabilitation exposure could not be performed. Fourth, disability was recorded as a binary clinical or administrative outcome rather than through standardized scales such as the modified Rankin Scale or Barthel Index, limiting sensitivity to functional changes. Accordingly, the disability analysis should be understood as an analysis of recorded disability status, not as a validated measure of post-stroke functional independence or activities of daily living [42]. Fifth, the dataset did not include dates of disability onset or certification; therefore, a valid cause-specific competing-risk analysis for disability with death as a competing event could not be performed. Sixth, some subgroup analyses were limited by sparse mortality and disability events, particularly in the intracranial stenting and embolization/coiling/other groups, leading to unstable or non-convergent models. For this reason, procedure-specific analyses were treated as exploratory and were not used for primary inference [25,43].

### 4.6. Future Directions

Future studies should employ prospective, multicenter designs that capture comprehensive clinical and rehabilitation variables. Observational longitudinal initiatives such as the IMPROVE study illustrate that rigorous follow-up at 3, 6, and 12 months with standardized assessments of motor function, cognition, mood, fatigue, and care situation is feasible and can provide valuable insights into long-term recovery after stroke [44]. Similar designs in middle-income settings should record key clinical measures including NIHSS, ASPECTS, occlusion site, reperfusion status, comorbidities (e.g., diabetes, kidney disease), complications (e.g., aspiration pneumonia, infection) and pre-stroke functional status because these factors strongly influence survival and disability [29]. Standardized functional outcome scales such as the modified Rankin Scale and Barthel Index are widely used worldwide, have good reliability and validity, and should be incorporated to allow comparability across studies. Combining these scales with newer instruments that assess participation (e.g., the Longshi Scale) may yield a more complete picture of patient outcomes [35].

To evaluate neurorehabilitation, future research should collect time-stamped data on rehabilitation initiation, session frequency, duration, intensity, and setting (inpatient vs. outpatient). Randomized trials and meta-analyses have suggested that very early and intensive mobilization (within 24 h) may not improve, and may even worsen, outcomes, whereas slightly delayed and moderate-frequency mobilization may offer benefit; however, evidence remains limited and contextual factors vary across health systems [45]. Prospective cohort studies with detailed rehabilitation data could apply time-dependent analytic methods and landmark analyses to identify optimal timing and dosing of rehabilitation interventions. Additionally, future research should explore sex-specific and social determinants of recovery. Earlier work shows that women often experience worse functional outcomes due to older age, higher pre-stroke disability and weaker social support [46]; capturing baseline functional status and social variables will allow examination of whether these patterns hold in middle-income contexts. Finally, collecting long-term follow-up data on participation, mood, cognition and quality of life will facilitate a more holistic understanding of recovery and inform interventions that address not only survival but also social reintegration and mental health after stroke [44].

## 5. Conclusions

In this retrospective cohort of adults who received advanced endovascular treatment for ischemic stroke in Almaty, older age and greater cumulative bed-day burden across intensive care, intermediate care, and rehabilitation wards during the index hospitalization were associated with a higher mortality hazard. Neurorehabilitation was associated with higher crude survival probabilities but not with time to death after multivariable adjustment. Because rehabilitation timing and baseline stroke severity were unavailable, this comparison does not provide an estimate of treatment effectiveness. The overall procedure term was not significantly associated with survival, and the lower mortality hazard observed for intracranial stenting in a pairwise comparison should be considered exploratory. Male sex was associated with binary recorded disability, but this administrative outcome was not a validated functional measure and was based on only 48 events. Prospective studies incorporating time-stamped rehabilitation exposure, stroke-severity measures, and standardized functional outcomes are required.

## Figures and Tables

**Table 1:** Baseline demographic, clinical, and hospital-utilization characteristics of patients undergoing high-tech care for ischemic stroke, stratified by survival status at end of follow-up and by disability outcome.

		Total	Living Status			Recorded Disability		
			Deceased	Alive	*p* Value	Disabled/Recorded	Not Disabled/Recorded	*p* Value
Age (mean ± SD)		63.38 ± 11.8	69.79 ± 9.3	61.20 ± 11.8	<0.001 ***	60.52 ± 10.5	63.87 ± 12.0	0.071
Sex	Male	61.6%	26.7%	73.3%	0.451	18.3%	81.7%	0.017 *
	Female	38.4%	23.0%	77.0%		8.7%	91.3%	
Place of living	Urban	91.2%	25.4%	74.6%	0.880	14.7%	85.3%	0.893
	Rural	8.8%	24.1%	75.9%		13.8%	86.2%	
Type of admission	Emergency Medical Service (EMS)	75.9%	28.5%	71.5%	0.035 *	14.1%	85.9%	0.802
	Self-Referral	18.3%	11.7%	88.3%		16.7%	83.3%	
	Primary Health Care	4.3%	21.4%	78.6%		21.4%	78.6%	
	Consultative and Diagnostic Care	0.3%	100.0%	0		0.0%	100.0%	
	Others	1.2%	25.0%	75.0%		0.0%	100.0%	
ICD-9-CM procedure codeICD-9-CM procedure code	00.65	31.1%	14.7%	85.3%	0.021 *	12.7%	87.3%	0.570
	39.72	2.1%	14.3%	85.7%		28.6%	71.4%	
	39.74	61.0%	30.5%	69.5%		14.5%	85.5%	
	39.75	5.8%	31.6%	68.4%		21.1%	78.9%	
Inpatient bed days (mean ± SD)		13.09 ± 7.7	15.62 ± 10.3	12.24 ± 6.4	<0.001 ***	13.52 ± 7.9	13.02 ± 7.7	0.683
Total bed days (mean ± SD)		3.64 ± 6	6.25 ± 8.4	2.75 ± 4.6	<0.001 ***	3.10 ± 5.3	3.73 ± 6.1	0.505
Time in days until death (mean ± SD)		855.38 ± 747.5	525.27 ± 758.7	967.21 ± 711.1	<0.001 ***	1263.10 ± 961.9	785.48 ± 682.2	<0.001 ***
Living status	Deceased	25.3%	-	-	-	15.7%	84.3%	0.759
	Alive	74.7%	-	-	-	14.3%	85.7%	
Disability	Disabled	14.6%	27.1%	72.9%	0.759	-	-	-
	Not Disabled	85.4%	25.0%	75.0%		-	-	-

Continuous variables are presented as mean ± standard deviation and were compared between groups using independent-samples t-tests. Categorical variables are presented as percentages and were compared using χ^2^ tests or Fisher’s exact test as appropriate. *p*-values < 0.05 indicate statistical significance. ICD-9-CM procedure codeICD-9-CM procedure code: 00.65—Percutaneous insertion of intracranial vascular stent(s); 39.72—Endovascular (total) embolization or occlusion of head and neck vessels; 39.74—Endovascular removal of obstruction from head and neck vessel(s); 39.75—Endovascular embolization or occlusion of vessel(s) of head or neck using bare coils *p* < 0.05 * and *p* < 0.001 ***.

**Table 2:** Characteristics and crude outcomes according to receipt of neurorehabilitation, including demographics, admission pathway, procedure code, and utilization measures.

			Frequency (%)	
Received neurorehabilitation		Yes	39%	
		No	61%	
		Received neurorehabilitation		
		Yes	No	*p* value
Age (mean ± SD)		62.57 ± 10.7	63.90 ± 12.5	0.323
Sex	Male	36.6%	63.4%	0.261
	Female	42.9%	57.1%	
Place of living	Urban	40.8%	59.2%	0.034 *
	Rural	20.7%	79.3%	
Type of admission	EMS	40.6%	59.4%	0.482
	Self-Referral	33.3%	66.7%	
	Primary Health Care	28.6%	71.4%	
	Consultative and Diagnostic Care	100.0%	0.0%	
	Others	50.0%	50.0%	
ICD-9-CM procedure code	00.65	35.3%	64.7%	0.445
	39.72	42.9%	57.1%	
	39.74	42.0%	58.0%	
	39.75	26.3%	73.7%	
Inpatient bed days (mean ± SD)		13.52 ± 6.4	12.82 ± 8.4	0.427
Total bed days (mean ± SD)		3.58 ± 4.8	3.67 ± 6.6	0.896
Time in days until death (mean ± SD)		852.57 ± 531.1	857.18 ± 859.1	0.952
Living status	Deceased	21.7%	78.3%	<0.001 ***
	Alive	44.9%	55.1%	
Recorded disability	Disabled/recorded	37.5%	62.5%	0.815
	Not Disabled/recorded	39.3%	60.7%	
Number of hospitalizations for neurorehabilitation	Mean ± SD: 0.19± 0.5	0.31 ± 0.7	0.12 ± 0.3	0.005 **

*p* < 0.05 *, *p* < 0.01 ** and *p* < 0.001 ***.

**Table 3:** Multivariable Cox proportional-hazards analysis of factors associated with time-to-death after endovascular intervention for ischemic stroke.

Variable	B	SE	Wald	df	*p*-Value	HR	95% CI for HR
Age, per year	0.067	0.011	37.192	1	<0.001 ***	1.069	1.046–1.092
Neurorehabilitation, yes vs. no	0.138	0.554	0.062	1	0.803	1.148	0.388–3.403
Number of neurorehabilitation hospitalizations	−0.591	0.335	3.115	1	0.078	0.554	0.287–1.068
Number of inpatient bed-days	0.002	0.018	0.009	1	0.923	1.002	0.967–1.038
Total bed-days	0.069	0.021	11.131	1	0.001 **	1.071	1.029–1.116
Male sex, vs. female	0.437	0.246	3.150	1	0.076	1.549	0.955–2.511
Procedure group	-	-	4.734	2	0.094	-	-
Intracranial stenting vs. mechanical thrombectomy/endovascular removal	−0.664	0.318	4.366	1	0.037 *	0.515	0.276–0.960
Embolization/coiling/other vs. mechanical thrombectomy/endovascular removal	−0.432	0.437	0.975	1	0.323	0.650	0.276–1.529

HRs > 1 indicate increased hazard of death, whereas HRs < 1 indicate decreased hazard of death. Continuous predictors are per-unit increases unless otherwise noted. The reference category for procedure type is mechanical thrombectomy/endovascular removal. Neurorehabilitation was entered as a fixed covariate because the dataset did not include dates of rehabilitation initiation or individual rehabilitation episodes. *p* < 0.05 *, *p* < 0.01 ** and *p* < 0.001 ***.

**Table 4:** Exploratory multivariable logistic regression analysis of factors associated with disability after endovascular intervention for ischemic stroke.

Variable	Model 1 OR (95% CI)	*p*-Value	Model 2 OR (95% CI)	*p*-Value	Model 3 OR (95% CI)	*p*-Value
Age, per year	0.977 (0.952–1.002)	0.077	0.974 (0.948–1.001)	0.055	0.974 (0.948–1.001)	0.069
Male sex, vs. female	2.339 (1.141–4.794)	0.020 *	2.305 (1.123–4.731)	0.023 *	2.662 (1.261–5.617)	0.010 *
Living status, dead vs. alive	-	-	1.345 (0.630–2.869)	0.443	1.381 (0.626–3.049)	0.423
Neurorehabilitation, yes vs. no	-	-	1.000 (0.519–1.925)	0.999	1.001 (0.514–1.949)	0.998
Number of inpatient bed-days	-	-	-	-	1.027 (0.976–1.081)	0.285
Procedure group	-	-	-	-	-	0.266
Intracranial stenting vs. mechanical thrombectomy/endovascular removal	-	-	-	-	0.668 (0.313–1.426)	0.296
Embolization/coiling/other vs. mechanical thrombectomy/endovascular removal	-	-	-	-	1.707 (0.589–4.948)	0.325
Total bed-days	-	-	-	-	0.939 (0.872–1.012)	0.092

Footnote: ORs > 1 indicate increased odds of disability, whereas ORs < 1 indicate decreased odds of disability. Continuous predictors are per-unit increases unless otherwise noted. The reference category for procedure type is mechanical thrombectomy/endovascular removal. Model 1 included age and sex; Model 2 additionally included living status and neurorehabilitation; Model 3 additionally included inpatient bed-days, procedure type, and total bed-days. *p* < 0.05 *.

## Data Availability

The datasets used and/or analyzed during the current study are available from the corresponding authors on reasonable request.

## Supplementary Materials

The following supporting information can be downloaded at: https://www.mdpi.com/article/10.3390/jcm15155964/s1. Table S1. Hierarchical logistic regression analysis of factors associated with final mortality status after endovascular intervention for ischemic stroke. Table S2. Cox proportional-hazards models for time-to-death by procedure group. Table S3. Logistic regression models for disability by procedure group.

## Abbreviations

The following abbreviations are used in this manuscript:

ANOVA	Analysis of variance
CI	Confidence interval
DALYs	Disability-adjusted life years
EMS	Emergency Medical Service
GCS	Glasgow Coma Scale
HR	Hazard ratio
ICD-9	International Classification of Diseases, Ninth Revision
ICD-10	International Classification of Diseases, Tenth Revision
ICU	Intensive care unit
IVT	Intravenous thrombolysis
LMICs	Low- and middle-income countries
MT	Mechanical thrombectomy
NIHSS	National Institutes of Health Stroke Scale
OR	Odds ratio
SD	Standard deviation
SMD	Standardized mean difference
SPSS	Statistical Package for the Social Sciences
