# Supplementary material for: Survival, Recorded Disability, and Neurorehabilitation After Advanced Endovascular Stroke Care in Kazakhstan: A Retrospective Cohort Study
Source: J Clin Med. 2026 Jul 30;15(15):5964. doi: 10.3390/jcm15155964 (PMC13467578; doi:10.3390/jcm15155964)
Supplement: Supplementary file 1 [file jcm-15-05964-s001.zip › jcm-4424943-supplementary.pdf]

Survival, Recorded Disability, and Neurorehabilitation After Advanced Endovascular Stroke Care in Kazakhstan: A Retrospective Cohort Study

Akerke Alibekovna Toleu [1,†], Mohammad Khoshraftar [2,†], Aizhan Bolatovna Almukhanova [3], Lyudmila Sergeevna Yermukhanova [1,*], Ardak Nurbakytovna Nurbakyt [4], Yesbol Tynysbekovich Toleu [5], Maral G. Nogayeva [6,*] and Alireza Afshar [1,2,*]

1. Supplementary

Table S1. Hierarchical logistic regression analysis of factors associated with final mortality status after endovascular intervention for ischemic stroke.

Variable	Model 1 OR (95% CI)	p-value	Model 2 OR (95% CI)	p-value	Model 3 OR (95% CI)	p-value
Age, per year	1.088 (1.056 -1.120)	<0.001***	1.087 (1.053 -1.121)	<0.001***	1.100 (1.064 -1.137)	<0.001***
Male sex, vs. female	1.540 (0.874 -2.713)	0.135	1.415 (0.788 -2.542)	0.245	1.752 (0.912 -3.366)	0.092
Disability, yes vs. no	-	-	1.469 (0.681 -3.171)	0.328	1.408 (0.618 -3.206)	0.415
Neurorehabilitation, yes vs. no	-	-	0.362 (0.197 -0.664)	0.001**	0.894 (0.256 -3.121)	0.861
Number of inpatient bed-days	-	-	-	-	1.040 (0.986 -1.096)	0.147
Total bed-days	-	-	-	-	1.073 (1.003 -1.146)	0.041*
Number of neurorehabilitation hospitalizations	-	-	-	-	0.525 (0.252 -1.092)	0.084
Procedure group	-	-	-	-	-	0.072
Intracranial stenting vs. mechanical thrombectomy/endovascular removal	-	-	-	-	0.424 (0.202 -0.889)	0.023*
Embolization/coiling/other vs. mechanical thrombectomy/endovascular removal	-	-	-	-	0.978 (0.314 -3.049)	0.970
Constant	0.001	<0.001***	0.001	<0.001***	<0.001	<0.001***

Footnote: ORs > 1 indicate increased odds of death, whereas ORs < 1 indicate decreased odds of death. Continuous predictors are per-unit increases unless otherwise noted. The reference category for procedure type is mechanical thrombectomy/endovascular removal. Model 1 included age and sex; Model 2 additionally included disability and neurorehabilitation; Model 3 additionally included inpatient bed-days, total bed-days, number of neurorehabilitation hospitalizations, and procedure type. $p<0.05$*, $p<0.01$** and $p<0.001$***.

Table S2. Cox proportional-hazards models for time-to-death by procedure group.

Procedure group	N	Deaths, n (%)	Model χ^2, df, p-value	Predictor	HR	95% CI	p-value
Mechanical thrombectomy/endovascular removal	200	61 (30.5)	$\chi^2 = 67.254$, df = 6, $p < 0.001$	Age, per year	1.062	1.035 - 1.089	<0.001***
Mechanical thrombectomy/endovascular removal	200	61 (30.5)	$\chi^2 = 67.254$, df = 6, $p < 0.001$	Neurorehabilitation, yes vs. no	0.776	0.219 - 2.754	0.695
Mechanical thrombectomy/endovascular removal	200	61 (30.5)	$\chi^2 = 67.254$, df = 6, $p < 0.001$	Number of neurorehabilitation hospitalizations	0.643	0.322 - 1.282	0.209
Mechanical thrombectomy/endovascular removal	200	61 (30.5)	$\chi^2 = 67.254$, df = 6, $p < 0.001$	Number of inpatient bed-days	0.969	0.917 - 1.024	0.271
Mechanical thrombectomy/endovascular removal	200	61 (30.5)	$\chi^2 = 67.254$, df = 6, $p < 0.001$	Total bed-days	1.097	1.036 - 1.161	0.001**
Mechanical thrombectomy/endovascular removal	200	61 (30.5)	$\chi^2 = 67.254$, df = 6, $p < 0.001$	Male sex, vs. female	1.491	0.846 - 2.624	0.166
Intracranial stenting	102	15 (14.7)	$\chi^2 = 11.992$, df = 6, $p = 0.062$	Age, per year	1.055	0.987 - 1.128	0.111
Intracranial stenting	102	15 (14.7)	$\chi^2 = 11.992$, df = 6, $p = 0.062$	Neurorehabilitation, yes vs. no	1.104	0.105 - 11.623	0.934
Intracranial stenting	102	15 (14.7)	$\chi^2 = 11.992$, df = 6, $p = 0.062$	Number of neurorehabilitation hospitalizations	0.784	0.157 - 3.901	0.766
Intracranial stenting	102	15 (14.7)	$\chi^2 = 11.992$, df = 6, $p = 0.062$	Number of inpatient bed-days	1.067	0.970 - 1.175	0.186
Intracranial stenting	102	15 (14.7)	$\chi^2 = 11.992$, df = 6, $p = 0.062$	Total bed-days	1.151	0.973 - 1.363	0.102
Intracranial stenting	102	15 (14.7)	$\chi^2 = 11.992$, df = 6, $p = 0.062$	Male sex, vs. female	5.893	0.664 - 52.300	0.111
Embolization/coiling/other	26	7 (26.9)	Model did not converge	-	-	-	-

Footnote: HRs > 1 indicate increased hazard of death, whereas HRs < 1 indicate decreased hazard of death. Continuous predictors are per-unit increases unless otherwise noted. Cox proportional-hazards models were fitted separately within each procedure group; therefore, procedure type was not estimated as a predictor within strata. Coefficients from non-convergent Cox models were not reported. p<0.05*, p<0.01** and p<0.001***.

Table S3. Logistic regression models for disability by procedure group.

Procedure group	N	Disability events, n (%)	Model χ^2, df, p-value	Predictor	OR	95% CI	p-value
Mechanical thrombectomy/endovascular removal	200	29 (14.5)	χ^2 = 13.230, df = 6, p = 0.040	Age, per year	0.965	0.929 - 1.001	0.055
Mechanical thrombectomy/endovascular removal	200	29 (14.5)	χ^2 = 13.230, df = 6, p = 0.040	Male sex, vs. female	2.230	0.884 - 5.625	0.089
Mechanical thrombectomy/endovascular removal	200	29 (14.5)	χ^2 = 13.230, df = 6, p = 0.040	Living status, dead vs. alive	2.303	0.854 - 6.211	0.099
Mechanical thrombectomy/endovascular removal	200	29 (14.5)	χ^2 = 13.230, df = 6, p = 0.040	Neurorehabilitation, yes vs. no	0.720	0.297 - 1.751	0.470
Mechanical thrombectomy/endovascular removal	200	29 (14.5)	χ^2 = 13.230, df = 6, p = 0.040	Number of inpatient bed-days	1.057	0.981 - 1.138	0.147
Mechanical thrombectomy/endovascular removal	200	29 (14.5)	χ^2 = 13.230, df = 6, p = 0.040	Total bed-days	0.906	0.823 - 0.997	0.046*
Intracranial stenting	102	13 (12.7)	Model did not converge	-	-	-	-
Embolization/coiling/other	26	6 (23.1)	χ^2 = 4.352, df = 6, p = 0.629	Age, per year	0.987	0.909 - 1.072	0.763
Embolization/coiling/other	26	6 (23.1)	χ^2 = 4.352, df = 6, p = 0.629	Male sex, vs. female	2.435	0.266 - 22.263	0.431
Embolization/coiling/other	26	6 (23.1)	χ^2 = 4.352, df = 6, p = 0.629	Living status, dead vs. alive	0.589	0.031 - 11.213	0.725
Embolization/coiling/other	26	6 (23.1)	χ^2 = 4.352, df = 6, p = 0.629	Neurorehabilitation, yes vs. no	0.477	0.032 - 7.214	0.593
Embolization/coiling/other	26	6 (23.1)	χ^2 = 4.352, df = 6, p = 0.629	Number of inpatient bed-days	0.907	0.693 - 1.185	0.474
Embolization/coiling/other	26	6 (23.1)	χ^2 = 4.352, df = 6, p = 0.629	Total bed-days	1.123	0.870 - 1.449	0.373

ORs > 1 indicate increased odds of disability, whereas ORs < 1 indicate decreased odds of disability. Continuous predictors are per-unit increases unless otherwise noted. Logistic regression models were fitted separately within each procedure group; therefore, procedure type was not estimated as a predictor within strata. Coefficients from non-convergent logistic models were not reported. $p<0.05$*, $p<0.01$** and $p<0.001$***.
